# RELATIONSHIP BETWEEN PHYSIOLOGICAL MEASURES AND COGNITIVE FUNCTION IN OLDER ADULTS WITH DEMENTIA

**DOI:** 10.1093/geroni/igae098.2595

**Published:** 2024-12-31

**Authors:** Derong Zeng

**Affiliations:** Kyoto University, Kyoto City, Kyoto, Japan

## Abstract

This study explores the interrelationships among physiological indicators in dementia patients, focusing on the correlation between Mini-Mental State Examination (MMSE) scores and indicators like blood pressure, heart rate, and body temperature, aiming to contribute new insights for early dementia diagnosis and treatment. Research involved 337 dementia patients, employing descriptive statistical analysis, correlation analysis, multivariate linear regression, and principal component analysis, with ethical approval from Kyoto University Hospital Ethics Committee (R3258). Findings reveal an average MMSE score of 21.69 (SD=5.33), highlighting a significant positive correlation between diastolic blood pressure (DBP) and MMSE scores (r=0.139, p=0.0030), while associations with systolic blood pressure, pulse rate, and body temperature were not significant. The first principal component accounted for 40.51% of variance, indicating that DBP may significantly impact cognitive function in dementia patients, suggesting blood pressure management’s potential in cognitive function maintenance. However, weak associations with other physiological indicators underscore dementia’s complexity, influenced by multiple factors.

